# Transcriptome profiling of *Saccharomyces cerevisiae* mutants lacking C2H2 zinc finger proteins

**DOI:** 10.1186/1471-2164-9-S1-S14

**Published:** 2008-03-20

**Authors:** Jinghe Mao, Tanwir Habib, Ming Shenwu, Baobin Kang, Wilbur Allen, LaShonda Robertson, Jack Y Yang, Youping Deng

**Affiliations:** 1 Department of Biology, Tougaloo College, Tougaloo, MS 39174, USA; 2 Department of Biological Sciences, University of Southern Mississippi, Hattiesburg, MS 39406, USA; 3 Harvard Medical School, Harvard University, Cambridge, Massachusetts 02140-0888 USA

## Abstract

**Background:**

The budding yeast *Saccharomyces cerevisiae* is a eukaryotic organism with extensive genetic redundancy. Large-scale gene deletion analysis has shown that over 80% of the ~6200 predicted genes are nonessential and that the functions of 30% of all ORFs remain unclassified, implying that yeast cells can tolerate deletion of a substantial number of individual genes. For example, a class of zinc finger proteins containing C2H2 zinc fingers in tandem arrays of two or three is predicted to be transcription factors; however, seven of the thirty-one predicted genes of this class are nonessential, and their functions are poorly understood. In this study we completed a transcriptomic profiling of three mutants lacking C2H2 zinc finger proteins, *ypr013cΔ,ypr015cΔ* and *ypr013cΔypr015cΔ*.

**Results:**

Gene expression patterns were remarkably different between wild type and the mutants. The results indicate altered expression of 79 genes in* ypr013*cΔ, 185 genes in *ypr015*cΔ and 426 genes in the double mutant when compared with that of the wild type strain. More than 80% of the alterations in the double mutants were not observed in either one of the single deletion mutants. Functional categorization based on Munich Information Center for Protein Sequences (MIPS) revealed up-regulation of genes related to transcription and down-regulation of genes involving cell rescue and defense, suggesting a decreased response to stress conditions. Genes related to cell cycle and DNA processing whose expression was affected by single or double deletions were also identified.

**Conclusion:**

Our results suggest that microarray analysis can define the biological roles of zinc finger proteins with unknown functions and identify target genes that are regulated by these putative transcriptional factors. These findings also suggest that both YPR013C and YPR015C have biological processes in common, in addition to their own regulatory pathways.

## Background

The budding yeast, *Saccharomyces cerevisiae,* has been an excellent eukaryotic model system for understanding basic cellular processes and metabolic pathways [1]. *S. cerevisiae* was the first eukaryotic genome for which the genome sequence was reported [2]. Approximately 6200 ORFs were identified; however, over 30% of the genes remain functionally unclassified. Furthermore, large-scale gene deletion analysis has shown that over 80% of the ~6200 yeast genes are nonessential, implying that many genes and pathways in this organism are functionally redundant [3,4].

Zinc finger proteins (Zfp) represent the largest and most diverse superfamily of nucleic acid binding proteins in eukaryotes. These proteins participate in a variety of cellular activities, including development, differentiation, cell cycle, and tumor suppression. It has been estimated that up to 1% of the genes in the human genome may encode proteins with zinc finger domains [5]. In the human brain alone, 133 species of C2H2 type zinc finger cDNAs have been identified [6-8]. Currently, ˜ 31 C2H2 zinc finger proteins have been reported and/or predicted to be transcriptional factors in yeast [9,10]. The functions of 24 zinc finger proteins have been extensively studied; however, the remaining seven genes (YER130C, YGR067C, YML081W, YPL230W, YPR013C, YPR015C and YPR022C) are nonessential with little or unknown biological functions (). YPR015C was recently identified as one of 100 novel, weakly expressed cell cycle-regulated genes when yeast were grown in a fermentor using minimum medium, indicating that some of the transcriptional factors may be not activated in rich medium [11]. Systematic genetic analysis revealed a synthetic lethal interaction between CTF4 and YPR015C, leading to an impairment of POL II transcription [12], suggesting that two genes may be involved in the same essential pathway. Limited information pertinent to nonessential genes exists in current scientific literature.

The *S. cerevisiae* deletion library contains deletions of all 4700 nonessential genes [3]. These mutants provide a valuable resource for genome-wide functional analyses. Transcriptomic analysis permits the simultaneous profiling of gene expression of thousands of genes and the identification of target genes regulated by specific gene of interest via mutation. We chose to study two non-essential genes, YPR013C and YPR015C, which are located on the same chromosome (chr XVI). These genes encode C2H2 zinc finger proteins with two Zfs in a tandem array, four identical stretches, and a conserved linker [10]. We examined the gene expression patterns of the two single deletion mutants, as well as a double mutant harboring both these gene deletions. It is our objective to understand how transcriptional regulation is affected by these particular zinc finger proteins, and to identify common features among various pathways of transcriptional regulation.

## Results and discussion

To investigate the biological roles of the two C2H2 zinc finger proteins, we chose to examine the effect of the two gene deletion on overall gene expression in the mutants. Microarray analyses of single mutant *ypr013c*Δ and *ypr015c*Δ macrodissect the target genes or regulatory pathways for those particular zinc finger proteins and the synergistic effect of the deletion of both Zf motifs by profiling of the double mutant *ypr013c*Δ*ypr015c*Δ.

### Identification of differentially expressed genes among two different single mutants and a double mutant

**Figure 1:**
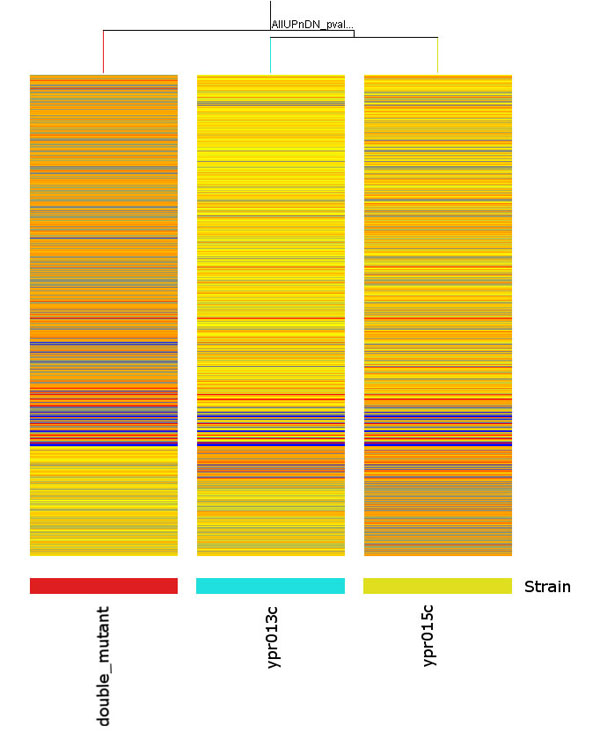
**Condition tree for all significantly UP and Down-regulated genes, total 562 genes**. Orange represents up and blue represent down-regulated genes.

Genome-wide expression profiles for two single deletion mutants (*ypr013c*Δ and *ypr015c*Δ) and a double mutant (*ypr013c*Δ*ypr015c*Δ) were generated using the 60-mer oligonucleotide array capable of detecting 6225 different yeast genes. Hierarchical Cluster Analysis (HCL) of the correlation matrix of the normalized microarray ratio data shows clustering according to the single and double mutants. The correlation of expression between and within samples suggests that gene expression patterns are different among the single and double mutants. The Welch T-test was used to determine significant changes in gene expression between mutants and wild type. A total of 562 genes were found to be differentially expressed with a cut-off ρ < 0.05 and fold change > 1.5. The expression pattern of the double mutant is more similar to that of the single mutant *ypr015c*Δ (Figure 1). Among the 562 genes, 337 genes were up-regulated and 225 genes were down-regulated when the gene expression ratios in the mutants were compared to those in wild type strains (Figure 1).

**Figure 2:**
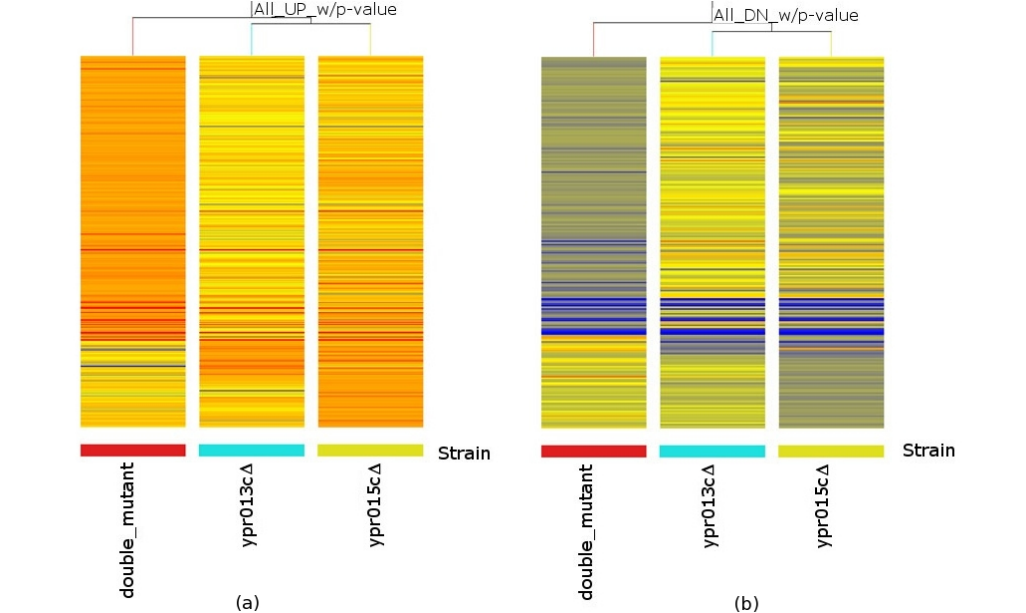
**Condition tree for all significantly UP and Down-regulated genes, total 562 genes**. (a), Significantly up-regulated genes are represents in orange, 337 genes. (b) Down-regulated genes are represents in blue, 225 genes.

The HCL results demonstrated that the samples on the same biological condition grouped together, indicating that the experimental quality was well controlled. Both *ypr013c*Δ *ypr015c*Δ and *ypr015c*Δ showed a greater degree of altered gene expression than *ypr013c*Δ (Figures 1, 2). For example, 258 and 104 up-regulated genes were present on the arrays of the double mutant and *ypr015c*Δ, respectively, whereas only 53 were present in *ypr013c*Δ. Similarly, there were 168 and 81 down-regulated genes in the double mutant and *ypr015c*Δ, respectively, and 26 genes in *ypr013c*Δ (Figure 1). This suggests that Ypr013cp may have less of an effect on transcriptional regulation than Ypr015cp in yeast cells. By comparing the extent of the gene expression changes caused by single and double deletion, it can be easily determined that the changes were significant. More than 80% of 562 altered gene expressions appeared in double mutants, with 206 genes up-regulated and 136 down-regulated. The double deletions greatly affected the transcriptomic profile and suggest synergistic effects on multiple biological pathways. For example, the two genes YDR342C/HXT7 and YDR343C/HXT6, which encode glucose transporters and are involved in glucose signal transduction pathways [13,14], were up-regulated to 3-4 fold in double mutants yet none in single mutants (Figure 1).

**Figure 3:**
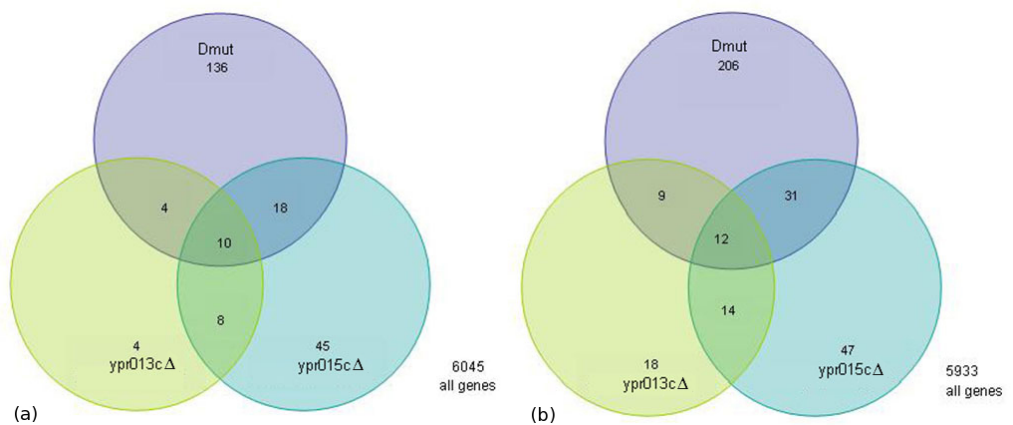
**Venn diagrams for genes whose expression was significantly altered in the single mutants and in the double mutant**. Green indicated genes whose expressions were altered in *ypr013c*Δ, blue in *ypr015c*Δ and violet in *ypr013c*Δ*ypr015c*Δ. (a), Overlapped up-regulated genes, and (b), Overlapped down-regulated genes.

**Figure 4:**
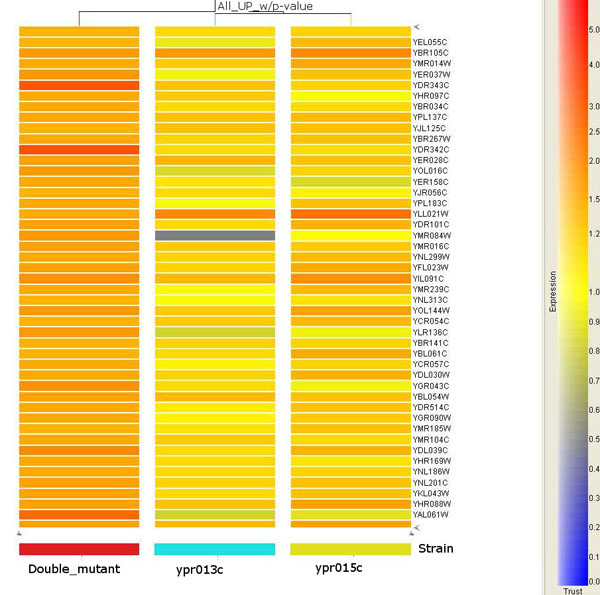
**Expression of HXTs can be detected in the double mutant**. Orange represents up and blue represent down-regulated genes. Two HXTs (in red color) are up-regulated by 3-4 folds in the *ypr013c*Δ*ypr015c*Δ strain.

**Figure 5:**
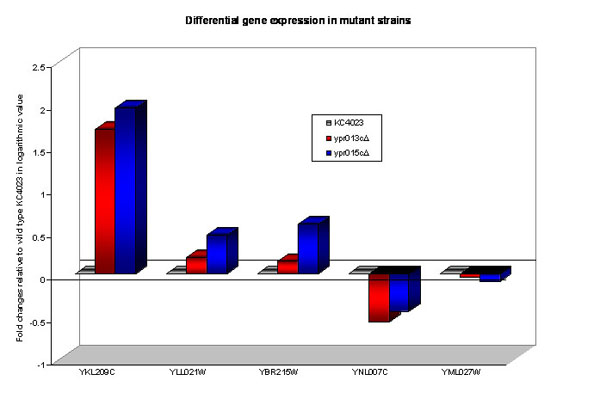
**Real-time PCR analysis for differentially expressed genes in the two single mutants**. Reactions were normalized to a stable housekeeping gene (actin), and fold changes were calculated relative to the wild type strain in logarithmic value. All reactions were performed in triplicate, and the average fold change is shown.

Analysis of differentially expressed genes held in common among mutants is a good indicator of relatedness. We used the abundance of genes in common among differentially expressed gene lists to identify the gene distribution (Figure 3). Among the 337 up-regulated genes, 12 common genes are observed in all the mutants (Table 1), 43 common genes in the double mutant and *ypr015c*Δ, 21 common genes in the double mutant and *ypr013c*Δ and 26 common genes in *ypr013c*Δ and *ypr015c*Δ. Among 225 down-regulated genes, 10 common genes are shown in all the mutants (Table 2), 28 common genes in the double mutant and *ypr015c*Δ, 14 common genes among the double mutant and *ypr013c*Δ and 18 common genes in *ypr013c*Δ and *ypr015c*Δ. Functional biological pathways often consist of multiple genes. MIPS analysis of twelve up-regulated and ten down-regulated common genes was performed to determine which gene classes were regulated similarly among the deletion mutants. The finding revealed enrichment of genes encoding proteins involved in metabolism, transcription, signal transduction and interaction with environment.

**Table 1:** Overlapped up-regulated gene list with fold change and p-value among the double mutant and single mutants.

Systematic Name	*ypr013CΔ*		*ypro015CΔ*		*ypr013CΔ/ypro015CΔ*	
	Fold change	p value	Fold change	p value	Fold change	p value
YNL308C	1.695	0.0302	2.007	0.0214	1.598	0.000585
YGR145W	1.725	0.0298	2.459	0.0426	1.735	0.00712
YBR105C	1.786	0.0211	2.02	0.016	1.729	0.0467
YFL026W	44.03	0.0129	39.28	0.0436	39.88	0.000223
YBR093C	2.138	0.0128	1.889	0.00896	3.13	2.40E-05
YAR071W	1.959	0.00896	2.369	0.00223	4.505	1.40E-05
YCR097W	10.53	0.00494	6.418	0.0364	9.095	0.00192
YKL209C	20.0	0.00443	22.29	0.00322	18.58	3.28E-05
YNL145W	9.411	0.00336	7.992	0.00394	9.132	6.87E-06
YIL015W	28.0	0.0022	22.14	0.00171	13.62	1.49E-05
YGL244W	1.546	0.000913	2.09	0.00151	1.547	0.0107
YDR461W	10.07	0.000397	9.539	0.0172	11.4	9.65E-07

**Table 2:** Overlapping down-regulated gene list with fold change and p-value among the double mutant and single mutants.

Systematic Name	*ypr013CΔ*		*ypro015CΔ*		*ypr013CΔ/ypro015CΔ*	
	Fold change	p value	Fold change	p value	Fold change	p value
YJR004C	0.0551	0.0323	0.0542	0.0212	0.0604	1.57E-05
YAR075W	0.46	0.0115	0.462	0.00555	0.616	0.000742
YOR237W	0.453	0.00987	0.368	0.00719	0.491	3.57E-05
YGL089C	0.016	0.00357	0.0126	0.000153	0.0206	6.70E-05
YCR040W	0.115	0.00357	0.112	0.0407	0.144	2.60E-08
YLR303W	0.012	0.00281	0.0113	0.000898	0.0147	9.53E-05
YCR097W-A	0.248	0.00193	0.22	0.0484	0.272	1.42E-06
YLR040C	0.119	0.00173	0.0957	0.00222	0.144	1.67E-05
YKL178C	0.0237	0.000644	0.0228	0.0107	0.0228	3.77E-09
YPL187W	0.01	0.000439	0.01	0.00029	0.0104	2.80E-12

### Confirmation of array results by real-time PCR

To validate the breadth of fold differences of microarray results, several ORFs were verified by real-time PCR (Figure 1). One of selected ORFs was YKL209C, which encodes the mitochondrial malic enzyme involved in sugar metabolism and was highly up-regulated (~ 20 fold) in all mutants. YLL021W encoding the spindle pole antigen and YBR215W involved in cell-cycle regulation were up-regulated only in the *ypr015c*Δ mutant (~ 2 fold). YNL007C, which is also involved in cell cycle regulation, was down-regulated in both single mutants (~ 2-3 fold), while YML027W also a cell cycle regulated gene was down-regulated in *ypr015c*Δ mutant alone (~ 2 fold). As shown in Figure 1, the expression patterns produced by real time PCR were similar with that of microarray analysis. Hence, the microarray findings were validated.

### Functional categories of the significant genes

Functional biological pathways often consist of multiple genes. The 562 differentially-expressed genes were categorized according to the Munich Information Center for Protein Sequences (MIPS). MIPS assigns some of the genes to more than one functional category, which is reflected in the distribution. By comparing the alteration in biological processes among all mutants, as shown in Table 3, distinct changes can be seen. These changes are related to effects of the different gene deletions. For example, the highest proportion (36-42%) of up-regulated genes belonging to the transcription category was present in *ypr015c*Δ and the double mutant, and the greatest percentage (38%) of genes belonging to the metabolism was up-regulated in *ypr013c*Δ. These results indicate that Ypr015cp primarily serves as a transcriptional repressor for a variety of genes whereas Ypr013cp plays a significant role in metabolism, in addition to its role in modulating transcription (29%). Other processes associated with these genes are cell rescue, defence, and virulence (14-20%). The genes in the pathways for stress responses, toxin and immune responses were mainly down-regulated, suggesting a decreased response to stress conditions. This is consistent with observations in which *ypr013c*Δ was sensitive to 37°C on YPD and *ypr015*cΔ showed slow growth on YPG at both 30°C and 37°C (data not shown). In addition, genes involved in the cell cycle and DNA processing are almost equally distributed between up-regulated (7-16%) and down-regulated (9-14%) genes in all mutants.

**Table 3:** Distribution of differentially expressed genes among the single mutants and the double mutants according to MIPS functional categories. Some of the genes are assigned to more than one functional category by MIPS.

Functional Category	Up-regulated			Down-regulated		
	*ypr013CΔ*	*ypro015CΔ*	*ypr013CΔ/ypro015CΔ*	*ypr013CΔ*	*ypro015CΔ*	*ypr013CΔ/ypro015CΔ*
Metabolism	38.1%	19.2%	20.4	24%	21.0%	34.8
Transcription	29.0%	35.9%	42.1	6.0%	9.2%	10.1
Protein with Binding	18.1%	26.3%	31.4	6.0%	9.2%	15.8
Protein Synthesis	3.63%	10.5%	14.1	2%	7.56%	12
Cell Cycle & DNA process	7.27%	15.7%	12.2	12%	9.24%	13.9
Cellular Transport	16.3%	12.2%	11	10%	8.4%	16.4
Energy	14.5%	6.14%		2.0%	7.56%	
Protein fate	12.7%	8.77%	11.8	6.0%	5.04%	10.7
Unclassified	9.09%	13.1%	11.8	40%	36.1%	14.5
Environment Interactions	20%	7.01%	11	20%	8.4%	10.7
Cell Rescue, Defense, Virulence	9.09%	7.89%	9.05	20%	14.2%	9.49

It is not unexpected that zinc finger protein deletions trigger an extensive altered expression of ~ 9% of protein encoding genes. As stated earlier, these proteins participate in a variety of cellular activities, including transcriptional control, development, differentiation, cell cycle and tumor suppression [6-8]. Ho *et al* identified six interactions between Ypr015cp and proteins in cell cycle regulation, cell rescue, metabolism by Affinity Capture-MS [15]; and Ptacek *et al,* using proteome chip technology, revealed 13 biochemical interactions in which Ypr013cp is involved [16]. Our findings are consistent with these data although determined using microarray analyses. Further study will be required to identify the promoters of target genes for Ypr013cp and Ypr015cp by CHIP on chip assay [17].

## Conclusions

We analyzed transcriptomic profiles in mutants lacking C2H2 zinc finger proteins by a combination of HCA and systematic functional analysis. Our data reveal that a single or a double deletion of YPR013C and YPR015C produced significant alteration of gene expression. The changes of gene expression induced by a double mutation, however, were more extensive, which may indicate synergistic effects on transcriptional regulation. Significant changes in functional categories were related to transcription, cell cycle regulation, and cell rescue. Our microarray results have provided the first genome-wide transcriptomic profiling to reveal the functional roles of two putative C2H2 zinc finger proteins.

## Materials and methods

### Yeast strains and plasmid

Isogenic *S. cerevisiae* wild type (KC 4023, same as BY 4741, *MAT*a *his3*Δ*1leu2*Δ *ura3*Δ* met15*Δ), *ypr013c*Δ, *ypr015c*Δ and *ypr013c*Δ *ypr015c*Δ were used in this study. The strains *ypr013c*Δ and *ypr015c*Δ are *MAT*a yeast deletion mutants, each carrying a gene deletion linked to a kanamycin-resistance marker *kanMX* that confers resistance to the antibiotic geneticin (G418). Wild type and single mutant strains were obtained from the Mississippi Functional Genomic Network Core facility. The double mutant was constructed by a PCR mediated gene disruption method [18]. Plasmid p4339 (pCRII-TOPO::natRMX4) serves as a DNA template to amplify the natRMX4 cassette required for PCR-mediated integration. Briefly, we used “fusion” PCR primers that contain 22 bp at their 3’ end, homologous to sequenced 5’ and 3’ of the natRMX4 cassette and 45 bp of either the 5’ or 3’end of the gene of interest. *ypr013c*Δ and *ypr015c*Δ were transformed with NAT^R^-ypr015c or NAT^R^-ypr013c fusion PCR products, respectively. Transformants were selected on YPD+G418 +clonNAT medium. Double mutants were confirmed by PCR (data not shown). All strains were grown to early log phase (1-2 X 10 ^6^ cells/ml) in YPD (1% yeast extract, 2% peptone, 2% glucose), then harvested for RNA preparation. Plasmid p4339 was kindly provided by the Mississippi Functional Genomic Network Core facility.

### Microarray hybridization

Total RNA was extracted from wild type, *ypr013c*Δ, ypr015cΔ and ypr013cΔ *ypr015c*Δ cultures grown to early log phase using TRIzol reagent according to the manufacture's instructions. The quality and quantity of RNA were measured by an Agilent 2100 bioanalyzer (Agilent, Palo Alto, CA). cRNA was synthesized by using a low-RNA-input fluorescent linear amplification kit (Agilent Technologies). Cy5 or Cy3 labeled cRNA was purified with the RNeasy MinElute kit (Qiagen, Valencia, CA, USA) and hybridized to yeast 60-mer oligonucleotide arrays according to the manufacturer's instruction (G4140B, Yeast V2, Agilent Technologies). Array slides were then scanned at 10Μm resolution with two-line averaging using an Axon GenePix 4200A scanner and GenePix 6.0 software. Microarrays were done in two (single mutants) or four (double mutants) replicate experiments, including both dye-swap technical replicates or/and biological replicates.

### Image and gene expression data analysis

Statistical analysis was done with Global locally weighted scatter-plot smoothing (LOWESS), Dye swap and ratio-based normalization. Scatter plot was used to identify the relationship between the two dyes and to check the hybridization quality. Log_2_ R is plotted against log_2_ G. Scatter plot is useful in early stage of analysis as it can help to determine whether a linear regression model is appropriate. A correlation between the variables results in the clustering of data points along a line. MA plot was also used to see the log-ratios and intensity-dependent effects at the same time. LOWESS regression, or locally weighted least squares regression, is a technique for fitting a smoothing curve to a dataset. It assumes that the dye bias appears to be dependent on spot intensity. Treatment and control channels are reversed in order to reduce the dye bias. Red and green dye intensity ratio is computed by:

log(R/G) -> log(R/G) – c(A)

where c(A) is the Lowess fit to the log(R/G) vs log(sqrt(R*G)) plot. Genes in *Ypr013CΔ* or *Ypro015CΔ* single mutant and double mutants (*Ypr013CΔ Ypro015CΔ*) were filtered based on flags present in four out of eight samples. Significant genes were selected with measure of confidence based on t-test, p-value. A cut off of ρ < 0.05 and fold change > 1.5 was used. Assuming there are false positives among the differentially expressed genes, we also used Benjamini and Hochberg false discovery rate (FDR) controlling approach [19]. We extracted 6045 quality genes for further data analysis for their functions using MIPS database. Genespring software (Agilent Technologies) was used to analyze microarray data.

### Real-time PCR

To validate the microarray data, a real-time PCR assay was performed to monitor gene expression in both wild type and mutant strains by comparing the mRNA levels of selected genes to a stable housekeeping gene (e.g., actin) using delta-delta Ct (threshold crossing value) calculations. cDNA was synthesized from 1 μg of purified RNA of the same samples used for cRNA synthesis for the microarray experiments by using Iscript cDNA synthesis kit (BioRad). Prior to use in real-time PCR, each primer set was validated for use by gel analysis of RT-PCR products. All reactions were performed in triplicate using a BioRad iCycler. Each reaction contained 12.5 ml SYBR green Supermix (BioRad, 100 mM KCl, 40 mM Tris HCl, pH 8.4, 0.4 mM of each dNTP, 0.5 U iTaq DNA polymerase, 6 mM MgCl2, 20 nM fluorescein), 0.5 ml forward and reverse primer (each at 5 mM), 1 ml cDNA, and H_2_O to a final volume of 25 ml. Reaction conditions were 1 cycle of 95°C for 1.5 min and 40 cycles of 95°C for 20 s, 60°C for 1 min.

## Competing interests

The authors declare that they have no competing interests.

## Authors' contributions

JM drafted the manuscript, contributed to the design of the study, and assisted with array analysis. TH analyzed microarray data and contributed to the writing of the manuscript. MS generated the double mutants and isolated total RNA from the mutants. BK conducted the array hybridizations and participated in the part of data analysis. WA and LR participated in the double mutant generation and part of data organization. YD directed this project and revised the manuscript. JYY helped to revise the manuscript. All authors approved the final manuscript.
